# Identification of Protein Complexes by Integrating Protein Abundance and Interaction Features Using a Deep Learning Strategy

**DOI:** 10.3390/ijms24097884

**Published:** 2023-04-26

**Authors:** Bohui Li, Maarten Altelaar, Bas van Breukelen

**Affiliations:** 1 Biomolecular Mass Spectrometry and Proteomics, Padualaan 8, 3584 CH Utrecht, The Netherlands; 2 Utrecht Institute for Pharmaceutical Sciences (UIPS), Utrecht University, Universiteitsweg 99, 3584 CG Utrecht, The Netherlands; 3 Mass Spectrometry and Proteomics Facility, The Netherlands Cancer Institute, 1066 CX Amsterdam, The Netherlands

**Keywords:** human protein–protein interaction, protein complexes, deep learning, data integration, proteomics, mass spectrometry

## Abstract

Many essential cellular functions are carried out by multi-protein complexes that can be characterized by their protein–protein interactions. The interactions between protein subunits are critically dependent on the strengths of their interactions and their cellular abundances, both of which span orders of magnitude. Despite many efforts devoted to the global discovery of protein complexes by integrating large-scale protein abundance and interaction features, there is still room for improvement. Here, we integrated >7000 quantitative proteomic samples with three published affinity purification/co-fractionation mass spectrometry datasets into a deep learning framework to predict protein–protein interactions (PPIs), followed by the identification of protein complexes using a two-stage clustering strategy. Our deep-learning-technique-based classifier significantly outperformed recently published machine learning prediction models and in the process captured 5010 complexes containing over 9000 unique proteins. The vast majority of proteins in our predicted complexes exhibited low or no tissue specificity, which is an indication that the observed complexes tend to be ubiquitously expressed throughout all cell types and tissues. Interestingly, our combined approach increased the model sensitivity for low abundant proteins, which amongst other things allowed us to detect the interaction of MCM10, which connects to the replicative helicase complex via the MCM6 protein. The integration of protein abundances and their interaction features using a deep learning approach provided a comprehensive map of protein–protein interactions and a unique perspective on possible novel protein complexes.

## 1. Introduction

Protein complexes are multi-protein assemblies that play a crucial role in diverse biological processes, including the control of cellular homeostasis, growth, and proliferation [1]. For example, the 26S proteasome, which consists of 31 different subunits, is essential in controlling the cell cycle, cell growth, and apoptosis by degrading obsolete or damaged proteins [2]. Elucidating the components and functions of multi-protein complexes is fundamental to understanding cellular processes. Despite tremendous efforts [3,4,5], it remains a daunting task to identify exactly which human proteins are present in protein complexes on a proteome-wide scale.

To identify protein complexes from protein–protein interactions, several experimental technologies are employed. For instance, yeast two-hybrid assays, which depend on bringing the DNA-binding domain (BD) and transcription activation domain (AD) of a eukaryotic transcription factor in close proximity by a bait-BD fusion protein and a pray-AD fusion protein, thereby enabling identification of protein interactions and protein complexes [6,7]. High-throughput experimental techniques, such as affinity purification–mass spectrometry (AP–MS) [8,9] and co-fractionation–mass spectrometry (CF–MS) [1,8] have enabled large-scale characterization of protein interactions. The AP–MS approach depends on the expression of a bait protein that is coupled to a matrix, allowing purification of the target proteins (preys) that interact with the bait from a lysate [10]. In the CF–MS approach, cellular lysates are extensively fractionated by multiple, non-denaturing biochemical methods that allow for the identification of protein complexes that co-elute [1]. Subsequently, a PPI network is represented by the co-elution network, and protein complexes are inferred using correlations of the protein elution profiles [1,11]. These high-throughput techniques have established the identification of large-scale protein interaction networks in humans and other model organisms, dramatically increasing the coverage of the PPI network.

In the past few years, two large-scale studies (BioPlex [5] and Hein et al. [8]) using the AP–MS approach, and one large-scale study by (Wan et al. [12]) using CF–MS have significantly improved the understanding of human PPI networks. However, the interactions identified by these different studies show only limited overlap [13]. One possible explanation may be that different experimental methods detect different types of interactions, thereby reporting different subsets of the actual PPI network [14]. Thus, Drew et al. integrated these datasets using a support vector machine (SVM) classifier to build a PPI network and ultimately obtained a global map of human protein complexes [13]. Besides these large-scale studies, many more protein–protein interaction datasets have been deposited into public repositories, such as BioGRID [15], BioPlex [16], and STRING [17]. This allows researchers to combine and integrate public datasets using in silico, e.g., computational approaches.

Proteins in a complex are typically expressed and localized in a spatiotemporal-similar manner, meaning that these proteins are often found in cellular vicinities simultaneously and possess similar biological functions [18]. Another predictor for protein interactions is to look at co-translation [2]. For instance, Shieh et al. showed that the proteins LuxA and LuxB are co-translated and assembled into the luciferase enzyme complex in *Escherichia coli* [19]. In addition, studies employing gene co-expression analyses have revealed that the network modules in a co-expression network are related to protein complexes. Examples of these complexes are the spliceosome, ribosome, and RNA polymerase II [20,21]. In addition, Bork and colleagues have constructed the STRING database [17], which incorporates data from multiple sources, including information on protein co-expression, text mining, and experimental data. This multi-level approach provides a system-wide view of protein–protein interactions [17,22,23], thereby showing the strength of data integration in the prediction of PPIs.

Although many efforts have been devoted to quantifying and classifying protein complexes, approaches by integrating large-scale protein abundance and interaction features need to improve. In this study, the integration of large-scale protein quantification data from multiple human cell samples was combined with AP–MS and CF–MS data to improve the construction of the human PPI network. We constructed a comprehensive map of human protein complexes via integrating protein interaction and protein abundance features. Briefly, the protein interaction features were obtained from three high-throughput AP–MS/CF–MS datasets [5,8,12], comprising 258 parameters describing different protein–protein interaction properties. The protein abundance features were derived from >7000 label-free human protein quantification samples from the PRoteomics IDEntifications (PRIDE) database (https://www.ebi.ac.uk/pride/, accessed on 20 June 2018). Subsequently, a deep learning (DL) model was built by using these features as input, and ultimately to infer an integrated protein interaction network. Next, a two-step unsupervised clustering procedure was performed to obtain a comprehensive map of human protein complexes. Our approach resulted in a comprehensive overview of protein complexes that also contain low-abundant and poorly characterized proteins, thereby providing a unique perspective on the human interactome.

## 2. Results

### 2.1. Feature Matrices Constructed by Incorporating Protein Abundance and Interaction Datasets

**Figure 1:**
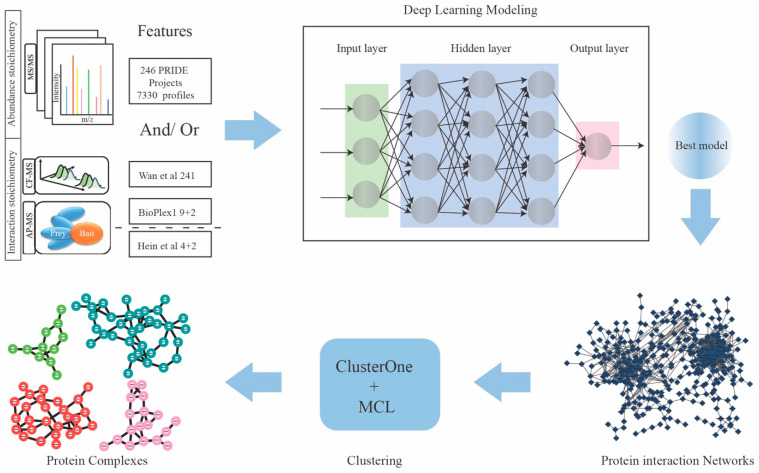
Flowchart for protein complex discovery. Schematic workflow for the discovery of protein complexes by employing deep learning algorithms. A total of 7330 protein quantification samples and three protein interaction datasets (Wan et al. [12], BioPlex [5], and Hein et al [8]. that contains 258 interaction features) were used as input to train the deep learning (DL) models; the optimal DL model was applied to infer protein–protein interaction scores and ultimately generated a weighted protein interaction network. Two unsupervised clustering algorithms, i.e., ClusterOne and MCL, were subsequently applied to obtain the final protein complexes dataset.

In this study, we integrated two recently published AP–MS protein interaction datasets from BioPlex and Hein et al., and one CF–MS protein interaction dataset [5,8,12]. As shown in Figure 1, we obtained 241 features from Wan et al.’s [12] CF–MS (co-fractionation–mass spectrometry) analysis of human proteins and their orthologues, comprising 6387 fractional MS experiments and over 999,000 interactions. Nine affinity purification mass spectrometry (AP–MS) features and two features generated by Drew and collaborators [13] were collected from BioPlex (Version 1) [5], which encompasses 2594 AP–MS experiments containing over 50,000 interactions from HEK239T cells. Four AP–MS features describing 28,504 interactions were obtained from Hein and colleagues [8].

**Figure 2:**
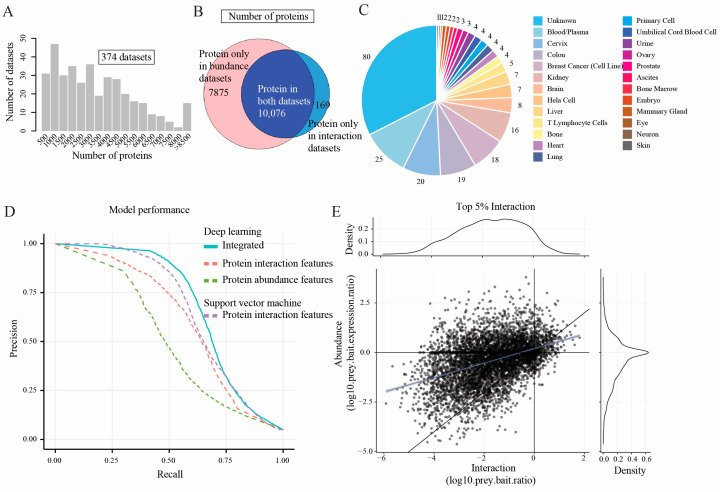
The integration of protein abundance and interaction features substantially improves model performance. (**A**) Distribution of the number of proteins in the protein abundance datasets. There are 374 datasets, and most datasets have quantified more than one thousand proteins. (**B**) A total of 17,951 and 10,245 proteins were collected from protein abundance samples and the protein interaction datasets separately, and over 98% of proteins (10,076 proteins) were observed in both datasets. The pink area represents the number of proteins only in protein abundance datasets, the light blue area represents the number of proteins only in protein interaction datasets, and the dark blue area represents the number of proteins in both datasets. (**C**) A pie chart showing the distribution of sample tissue specificities for the protein abundance samples. This plot shows that the protein abundance samples were distributed over more than 25 different tissues or organs, indicating a large sample diversity, which in turn improved the robustness of the deep learning model. Different colors indicate the organs, and the numbers in the pie chart are the numbers of datasets that are collected in the organs. Those datasets that do not show organ information in the PRIDE database are labeled “unknown.” (**D**) A comparison of model performance for the deep learning and SVM models based on different data sources. The blue line represents the best DL model using integrated protein abundance and protein interaction features, the red dashed line represents the best DL model using protein interaction features, the green dashed line is the best DL model using protein abundance features, and the purple dashed line represents the best SVM model using the protein interaction features. During the model training process, a list of protein–protein interaction (PPI) scores was predicted based on the test set and used to calculate the precision and recall by the “performance” function in the ROCR package in R. The precision is calculated by true positive/(true positive + false positive), and the recall is calculated by true positive/(true positive + false negative). The harmonic mean of precision and recall, namely the F1-measure or F1-score, was further used to determine the model performance. The integration of both abundance and interaction features (blue line) outperforms all other single feature based models (dashed lines). (**E**) A scatterplot showing the top 5% of protein interactions. From this plot it can be observed that the predicted protein–protein interactions greatly overlapped with the Hein et al. interaction network and exhibited similar distributions.

In PPI studies, researchers expect to retrieve subunits of complexes in equimolar amounts after immunoprecipitation (IP) from biological experiments. However, in practice, the range of detected interacting protein abundances spans several orders of magnitude [24]. This is caused by the possible involvement of some protein subunits in multiple different complexes with fractions of their total cellular pools, and subunits may behave differently under different states (different tissue or disease states). To reduce the bias caused by the huge span of protein abundances in protein complex identification, we incorporated >7000 protein abundance samples from the PRIDE archive [25]. Precisely, the protein abundance datasets were obtained from 246 quantitative proteomics projects, consisting of 7330 samples (Figure 1). The number of unique proteins detected in each sample ranged from 500 to over 8500 (Figure 2A). In total, we incorporated 17,951 proteins from protein abundance samples, which covers more than 98% of the proteins quantified in interaction datasets (Figure 2B). Moreover, the protein abundance samples were distributed over 25 different human tissues and organs, indicating a large sample diversity in our dataset (Figure 2C).

### 2.2. Model Performance Comparison

Having established the feature matrices, we next generated the training set and test set by labeling protein pairs based on a gold-standard literature-curated set of human protein complexes, CORUM [26]. The positively labeled protein–protein interactions (PPIs) are proteins within the same complex in the CORUM database. The negative protein pairs are those that are observed in the gold-standard set but that do not interact with subunits in the CORUM complexes. Protein pairs that were not included in the training process were labeled as “unknown”. Next, we implemented a deep learning neuronal network to train three types of models: (i) models using protein abundance features, (ii) models using protein interaction features, and (iii) models using integrated protein abundance and protein interaction features (Figure 2D). Moreover, to compare the performance of our models, we also built SVM models using the protein interaction feature matrix [27,28].

To obtain an optimal classifier, we trained our DL models by varying the number of neurons in three densely connected layers and the probabilities in dropout layers (details in Table S2). In addition, to decrease the impact of imbalanced datasets, we utilized the F1 measure and precision-recall curve as evaluation metrics to determine the performance of our models. This approach is widely recognized and has been applied in numerous studies that involve imbalanced data, including gene regulation network prediction and protein–protein interaction network prediction [13,29]. Our training process resulted in 1995 protein interaction feature-based models, where 63 models had an F1-measure > 0.59 (Figure S2A and Table S2A); 1921 protein abundance feature-based models, where 87 models had an F1-measure > 0.49 (Figure S2B and Table S2B); and 2338 integrated models (integrated protein abundance and interaction features), where 109 models had an F1-measure > 0.66 (Figure S2C and Table S2C) (see methods for details on the F1 measure). Moreover, 28 SVM classification models, based on protein interaction features, were obtained using a grid search algorithm (see methods). The precision-recall curve for the best models using the different feature matrices shows that the integrated deep learning model (Figure 2D, blue line, F1-measure = 0.68) outperformed all other models (F1-measure of protein abundance-DL, protein interaction-DL, and protein interaction-SVM models were 0.51, 0.61, and 0.64, respectively) (Figure 2D). The receiver operator characteristic (ROC) curve (Figure S1) presents similar results, where the area under the curve (AUC) for the integrated model is 0.9.

The optimal deep learning model contains 350, 140, and 25 neurons in three hidden layers, where dropout rates are 0.438, 0.214, and 0.037, respectively. It takes around 1.8 h to train the model. This model was further applied to predict the interaction score for all protein pairs characterized in the feature matrix. The optimal model takes ~80 s to make prediction for 10,000 protein pairs. The interaction score of a protein pair indicates the likelihood of that pair of proteins participating in the same complex. Subsequently, a weighted PPI network was generated, where the weights of edges were defined by the predicted interaction score (Figure 1). To assess the predicted PPI network, we further compared it with the network generated by Hein et al. (Figure 2E and Figure S3). Notably, the network formed by the top 5% of predicted interactions showed similar distributions to the Hein et al. network (Figure 2E) [8]. In addition, a weaker interaction was observed when the network was filtered by decreasing the protein interaction confidence, suggesting that a filtering step is required to obtain an optimal PPI network to infer protein complexes (Figure S3).

### 2.3. Protein Complexes Identified by Two-Stage Clustering Method

**Figure 3:**
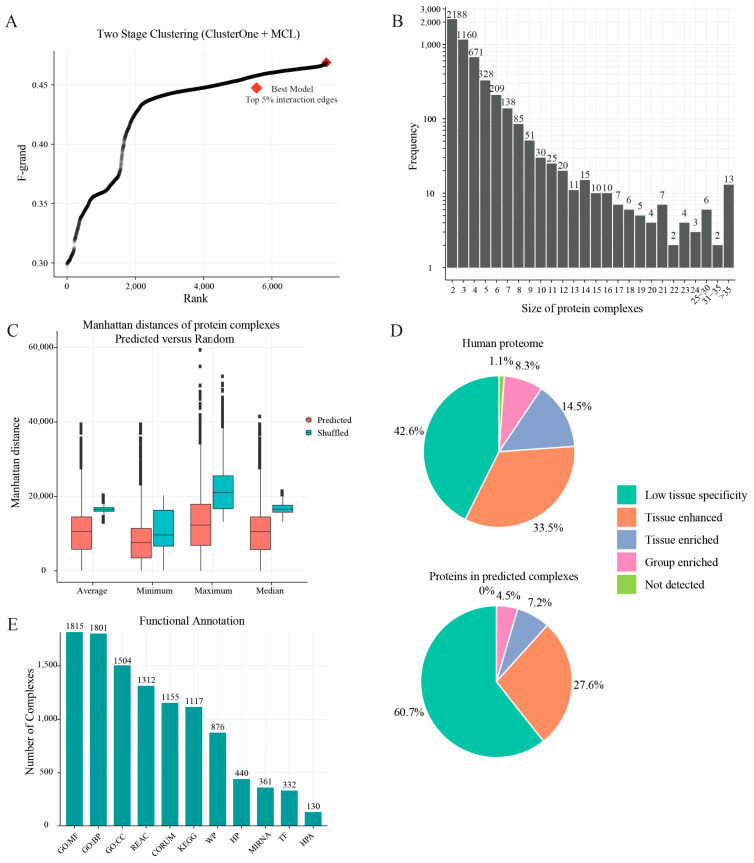
Biological features of predicted human protein complexes. (**A**) Parameter optimization for two-stage clustering (ClusterOne followed MCL) procedures. Each data point indicates one F-grand measure generated in the clustering step. This two-stage clustering step generated 7605 results with 3187 F-grand (points) over 0.45, indicating the high stability of the protein–protein interaction network. (**B**) Distribution of protein complex sizes in the final interaction map; the vast majority of protein complexes contain a small number of protein members. (**C**) Boxplots showing the average, minimum, maximum, and median of the protein complexes’ Manhattan distance as calculated based on the abundance of the protein complex subunits. The shuffled protein complex distance (blue) was evaluated by permuting protein members while maintaining the sizes of the protein complexes. It can be seen that the predicted complexes display a shorter Manhattan distance than the shuffled complexes, indicating the credibility of predicted protein–protein interactions. (**D**) Pie charts showing the proportions of proteins with varying tissue expression patterns from the Human Protein Atlas. From this plot, 60.7% of proteins in our complexes showed a low tissue specificity, indicating the ubiquitous expression property of the proteins. (**E**) The distribution of number of protein complexes with significantly enriched annotation terms using g: Profiler web tool. Most complexes could be enriched in one or more categories with significant terms, indicating the biological significance of complexes.

To elucidate the relationships among densely connected regions of the interaction network, a two-stage clustering was employed [13]. At the first stage of clustering, the ClusterOne algorithm [30] was employed to derive the intermediate clusters. Due to the appearance of over-merged clusters (merging high-overlapping clusters may lead to biologically unrelated complexes being merged) [12], we applied a second stage of clustering that is based on MCL [31] (see methods) to further break over-merged clusters produced by ClusterOne. To optimize the clustering performance, as described in the methods, we tuned the parameters including the top percentage of interaction edges r, density, and overlap parameters in ClusterOne, and inflation in MCL. A set of protein complexes resulting from each combination of parameters was compared with the gold-standard CORUM complex set by the k-clique algorithm [13], enabling the evaluation of their similarity and overlap to a benchmark complex set (here the CORUM complexes) on a global level. This two-stage clustering step generated 7605 datasets containing complexes, with their corresponding similarity measurements (F-grand values) as defined by the k-clique algorithm (Figure 3A). The best parameter combination was edges r: 119,560; density: 0.2; overlap: 0.8; and inflation (-I): 5, which resulted in an F-grand at 0.46. The optimal set contained 5010 complexes with 101,818 interactions (5% interaction edges of the full network) among 9129 human proteins (Table S4). Additionally, in line with the finding by Huttlin et al. [5], a vast majority of complexes contained a limited number of protein members (Figure 3B).

If a group of proteins can form a protein complex, we assumed that their expression might show a concordance, which could be evaluated by the Manhattan distance of their expression abundances. To evaluate the quality of the final predicted complexes further, we first evaluated the expression concordance of protein members in complexes by calculating the pair-wise Manhattan distance of the proteins using the abundance (label-free quantification) data. Subsequently, we randomly shuffled the protein members amongst the other complexes while maintaining the same complex size, and then calculated the Manhattan distance of proteins within these randomly generated complexes. This shuffling process was repeated 100 times. As indicated in Figure 3C, our final set displayed a shorter Manhattan distance within the complex than the shuffled complex set. Additionally, we annotated proteins with information on their expression in different tissues using the Human Protein Atlas [32]. We observed that a considerably high percentage of proteins in our complexes showed a low tissue specificity. For instance, the protein complex 76 (Table S4 line 76) that we predicted is reported as the proteasome [33] and the predicted complex 55 (Table S4 line 55) is known as the mediator complex [34], indicating that our complex set can capture many common fundamental processes in human cells (Figure 3D) [8].

Moreover, the functional annotation analyses of the protein complexes showed that a large proportion of predicted complexes could be enriched in functional terms (Figure 3E). For example, around 36% of the predicted complexes were significantly enriched in GO molecular functions and GO biological processes.

### 2.4. Protein Abundance Feature Contributes to Capturing Novel Subunits

**Figure 4:**
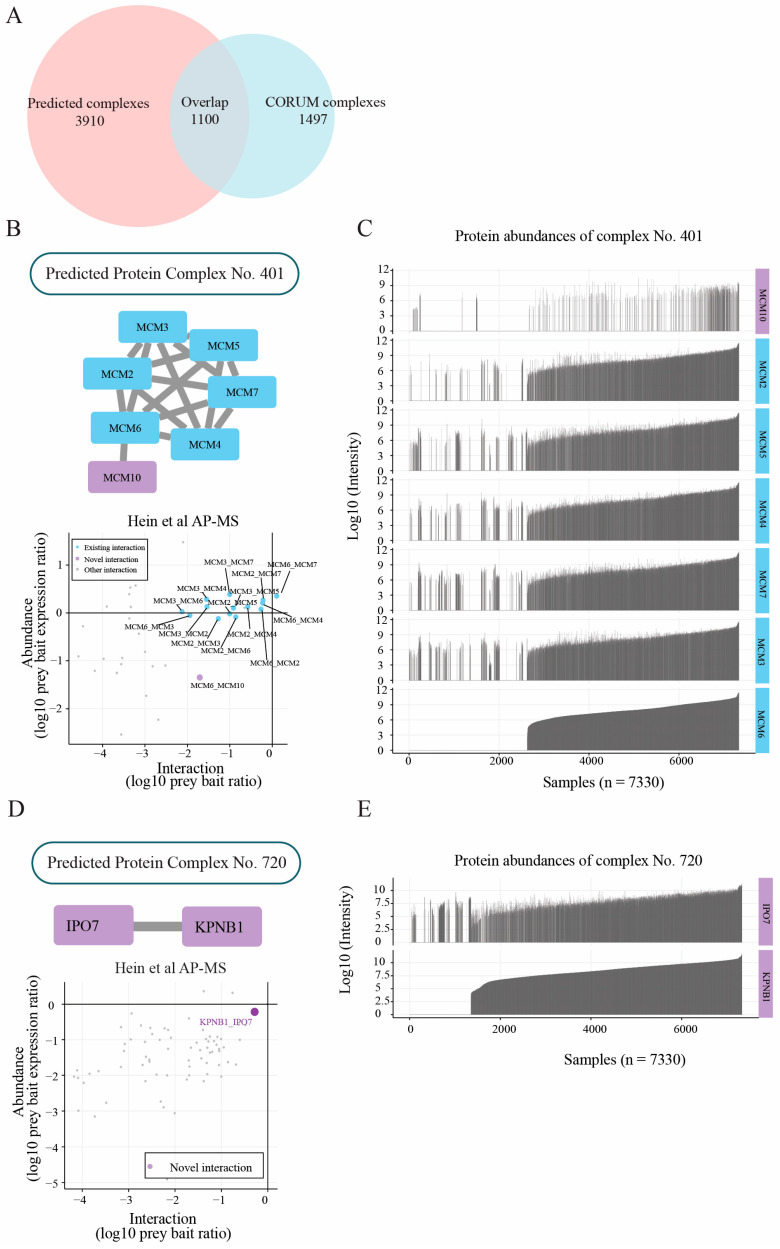
Selected complexes in the map contain novel subunits. (**A**) Venn plot indicating the overlap between the protein complexes predicted by our model (pink circle) and the complexes in CORUM database (blue circle). A total of 1100 out of 5010 predicted complexes exhibited a complete or partial overlap with the gold-standard protein complexes from the CORUM database, showing the potential to predict novel protein–protein interactions. (**B**, **top panel**); interaction network of replicative helicase; blue rectangles are known members of the MCM complex; and the purple rectangles are novel subunits as predicted by our deep learning model. (**B**, **bottom panel**); scatterplot with interaction and abundance features for the MCM complex from Hein et al.’s AP–MS experiments. Blue dots are known interactions, and the purple dots are novel interactions. Labels for the dots are represented by bait prey proteins. It can be observed from the scatterplot that the MCM6–MCM10 interaction follows a similar trend as the other known interactors, indicating that MCM10 could be a transient member of the MCM complex. (**C**) The expression pattern of each subunit within the MCM protein complex. On each row, the *X*-axis indicates 7330 samples collected from PRIDE repository, and the *Y*-axis indicates the protein abundance with corresponding protein name on right side, where missing values are in blanks. It can be observed that MCM10 is detected in fewer samples compared with the other subunits of the MCM complex, indicating a lower abundance or poor characterization potential by MS-based techniques. (**D**, **top panel**); interaction network of the new complex IPO7–KPNB1. (**D**, **bottom panel**); interaction-abundance plot for IPO7–KPNB1complex using Hein et al.’s AP–MS interaction data. This novel interaction was not observed in Hein et al.’s AP–MS interaction network data, demonstrating the sensitivity of our deep learning model. (**E**) The expression pattern of each subunit within the IPO7–KPNB1protein complex. The *X*-axis indicates 7330 samples collected from PRIDE repository. The *Y*-axis indicates the protein abundance with corresponding protein name on right side; missing values are in blanks. IPO7 and KPNB1 show significant co-expression in a majority (~5800) of the abundance samples, indicating a possible protein–protein interaction between IPO7 and KPNB1.

Our final dataset achieved a high model performance based on the F1-measure (0.68) and k-clique evaluation (F-grand = 0.46). In addition, approximately 15% (737 of 5010) of our complexes exhibited a complete or partial overlap with 42% (1100 of 2597) of the gold-standard complexes from the CORUM database (Figure 4A) [8]. This high confidence allowed us to predict novel interactions on top of known PPIs. For instance, we predicted MCM10 as a novel member of the MCM2–7 complex via interacting with MCM6 (Figure 4B). Furthermore, the protein abundance showed that the core subunits of the MCM2–7 complex exhibited a considerably high expression concordance in most of the 7330 samples we obtained from Pride (Figure 4C). Interestingly, MCM10 was detected in fewer samples, indicating its lower abundance or poor characterization potential by MS-based techniques. Based on these observations, we asked whether the MCM6–MCM10 interaction was detectable in AP–MS experiments. Indeed, the interaction data from Hein et al. [8] (Figure 4B, bottom) showed that the MCM6–MCM10 connection was detectable, although with a relatively low interaction, suggesting that MCM10 may be a non-obligatory or transient member of the MCM2–7 complex. In addition, Homesley et al. [35] and Douglas et al. [36] reported that MCM10 was required for the initiation of eukaryotic DNA replication, and physically interacts with MCM2–7 via subunit MCM6.

### 2.5. Members of Protein Complexes Exhibit Co-Expression Characteristic

Co-expression characteristics are of biological interest since co-expressed genes usually are controlled by the same transcriptional regulatory program, functionally related, or members of the same protein complex [37]. Proteins that are part of the same protein complex often show co-expression properties, and clusters of proteins with related functions often exhibit expression patterns that correlate under diverse conditions. For instance, importin-7 (IPO7) and importin beta-1 (KPNB1) are two important proteins for nuclear protein import [38]. These two proteins are highly co-expressed in a majority (~5800) of the abundance samples (Figure 4E). Moreover, the interaction between these two proteins was also detected in Hein et al.’s interaction network as a stable interaction (Figure 4D). Jakel et al. [39] reported that importin-7 (IPO7) and importin beta-1 (KPNB1) work as a heterodimer that binds to histone H1. (More examples showing co-expression properties are shown in the Supplementary Materials).

## 3. Discussion

Many vital cellular functions, including DNA replication, RNA transcription, and protein translation and regulation, require the coordination of proteins assembled into complexes. Thus, the analysis of protein complexes and PPI networks are of central importance in biological research. In the past decades, the combination of affinity purification/co-fractionation and mass spectrometry has advanced our understanding of protein complex composition. Increasing efforts have been devoted to generating larger-scale human protein interactions by integrating different AP–MS and CF–MS studies, and more comprehensive maps of protein complexes have been established [13]. Although these protein–protein interaction experiments are very well controlled studies, they are typically performed on certain cell lines/types and may overlook the proteomic abundance differences in human tissues. Here, we present a data integration method, using machine learning and classification algorithms, to create a comprehensive map of protein complexes by integrating protein interaction features and large-scale protein abundance samples.

In this work we developed a deep learning framework that incorporates multiple sources of data to establish a comprehensive human protein complex map. Our results show that a deep-learning-based approach, incorporating multiple sources of features (AP–MS/CF–MS interaction features and MS/MS protein abundance features), outperformed models using either interaction features or abundance features alone. In addition, these integrated deep learning models exhibit high robustness, not only on the F1-measure but also on the number of outperformed models (Section 2.2). We also showed that many complexes, including gold-standard and novel complexes, feature a unique characteristic of co-expression patterns in a majority of quantitative proteomics samples. This characteristic enabled us to recapitulate several well-known complexes, for instance the multi-synthetase complex [40] and eukaryotic initiation factor 2B complex [26] (Figure S5). Moreover, this characteristic also led us to discover highly co-expressed complexes, such as the IPO7–KPNB1 heterodimer complex (Figure 4D) and the VCP–HSPB90B1 complex (Figure S4E). These examples indicate that the expression levels of protein complex subunits are generally co-varying [18]. Thus, such co-varying characteristics can be used as one of the features for identifying protein–protein interactions and protein complexes. In contrast to other published methods, we did not summarize the concordance of protein expression between proteins as the correlation coefficient, as this may over simplify the complexity within a large dataset. Instead, we first calculated the expression difference within each protein pair among all 7330 protein abundance samples and subsequently used a deep learning algorithm to achieve a high-level featurization after training our model. Here, the state-of-art deep learning algorithm addresses this featurization by computing increasingly more complex features and then taking the results of preceding operations as input [41]. Therefore, our model makes full use of not only the protein interaction but also the protein abundance features with tissue/sample-level details.

In addition to model performance, the contribution of features is an important aspect of deep learning. Thus, we performed feature importance evaluation by the decrease of model performance by randomly shuffling the values of each feature (see Methods). As expected, the top-ranked features were the interaction features. For example, all of the top 15 features were interaction features, including “hein_neg_ln_pval”, “neg_ln_pval”, “hein_pair_count”, and “prey-bait correlation” (Figure S6B), which are the most important outcomes in AM–MS experiments. We also found that, within the protein abundance features, the feature importance was positively correlated with the number of proteins (Figure S6B). In other words, if more proteins are identified in a protein abundance sample, a higher importance that feature (i.e., protein abundance sample) shows. This suggests that the number of proteins in a protein abundance sample could be one of the criteria to improve the quality of the data in future works.

The weak interactions have frequently been overlooked or remained undetected, and they have been thought to be less important in large-scale interaction research, even though they are crucial features of networks in general [42,43]. In addition, weaker interactions with low abundant proteins are challenging to detect in AP–MS experiments [8]. To detect low-abundant proteins and characterize weak interactions, one possible strategy is to improve the sensitivity and resolution of the mass spectrometer or to remove high-abundant proteins from proteomic samples [44]. Another strategy is to increase data diversity via incorporating multiple sources of quantification samples, which is what we did in this study. The integration of the protein abundance samples and large-scale AP–MS experimental interaction networks enabled us to fill in the missing features caused by a single AP–MS experiment. For instance, we observed that the protein MCM10 binds to the MCM2–7 complex via MCM6 in a potential transient manner.

Overall, we observed a good performance of our model; however, there is still room for future improvements. Firstly, we included only the protein pairs with both interaction and abundance features to predict the PPI network. Ideally, the number of detected proteins accumulate and would ultimately reflect the total number of proteins in the human proteome when enough proteomic quantification features are collected. However, due to technical challenges, there is no such set of protein interaction studies that contains a comprehensive list of the whole proteomic-level baits. The lack of complete datasets limits the comprehensiveness of interaction features of protein pairs. The model based on these incomplete features therefore predicts an incomplete PPI network, which probably results in an incomplete protein complex map. Due to a lower performance of the deep learning model using only the protein abundance features, an expediency can be as follows: the core PPI network can be predicted using the integrated model, while the peripheral network is populated only using the protein abundance features model. Then the inference of the protein complex map based on this integrated core–peripheral network needs to be further explored. Secondly, protein structural information has been proved to be significant evidence for predicting PPI [45], which can be one more layer of information to be included in the future for improving the model performance. Thirdly, we found that support vector machines (SVM) outperformed deep learning (DL) when using only protein interaction features. We attempted to train the SVM models with integrated features or protein abundance features using a grid search to find the optimal combination of hyperparameters, C and gamma. To speed up the hyperparameter optimization process, we used multithreading by submitting 50 Slurm jobs to 50 nodes of the high-performance computing (HPC) system. However, due to the large number of dimensions in the dataset, none of the jobs was able to finish within the three-week maximum running time we had set. Therefore, feature engineering and selecting is a necessary step for the SVM model training. In addition, integrating protein interaction features specifically designed for deep learning algorithms may enhance their ability to classify protein interactions. By incorporating such features, the deep learning algorithms may be able to identify patterns and relationships within the protein interaction data better, leading to improved performance in predicting protein interactions. Finally, wet-lab experiments such as co-immunoprecipitations and more targeted approaches such as knockout studies need to be performed to validate and confirm the complexes further. However, according to the evaluation metrics of the deep learning model and protein complex map, we are convinced that the integration of the protein interaction features and the protein abundance features can improve the model’s performance, compared with using either type of these features alone. Thus, our work provides a new methodology to improve the reconstruction of PPI interaction and the understanding of protein complexes.

In conclusion, by incorporating interaction and large-scale protein abundance features, our deep learning framework serves as a pioneering protein complexes discovery analysis.

## 4. Materials and Methods

### 4.1. Gold-Standard Reference Set and the Training and Test Protein Pairs

A fundamental step in predicting protein complexes is the prediction of protein–protein interactions, which is considered a classification task in machine learning and requires a gold-standard reference set comprising a positive and a negative subset. The human protein complexes in the CORUM database [26] form a high confidence set of manually curated protein complexes and therefore can be considered as a gold-standard reference set in this study. The training and test sets that contain the gene names of protein pairs were downloaded from the hu.MAP database [13]. These training and test protein pairs were generated as described by Drew et al. [13] and were derived from the CORUM database. Briefly, a set of non-redundant complexes were retained by merging the complexes with a large overlap (i.e., Jaccard coefficient > 0.6) within the entire CORUM database. These non-redundant complexes were randomly divided into two sets, i.e., a training set and a test set. To ensure that the test and training sets of complexes were disjoint, complexes in the training set that shared any edge with a complex in the test set were removed. Next, the positive subset was defined by the set of protein pairs that were within the same complex. In contrast, the negative subset was defined by the set of protein pairs that were within the entire set of protein complexes but that were not in the same complex. To not skew the measurements of the performance of our model in subsequent classification steps, complexes with more than 30 subunits in the test set were removed. The positive and negative subsets of protein interactions were generated for both training and test sets, followed by removing the interactions from the training set that overlapped with those in the test set, such that the sets were fully disjointed. The final training set contained 14,186 and 95,802 protein–protein pairs in the positive and negative subsets, respectively. The test set contained 5781 and 111,055 protein–protein pairs in the positive and negative subsets, respectively.

### 4.2. Featurization of Protein–Protein Interaction Pairs

#### 4.2.1. Protein Abundance Features

We retrieved 246 independent projects from the PRIDE repository (Table S1 Projects information from PRIDE), containing a total of 374 datasets. All of these datasets were analyzed by MaxQuant [25]. The LFQ intensities from MaxQuant were used as the source of protein abundance features in this study. Within each dataset, reliable proteins were retained by removing potential contaminants and removing proteins that were identified with fewer than 2 peptides. In addition, the intensities of the proteins were averaged if one protein was reported in different dataset. Then we combined all datasets into one file by collecting all proteins and samples. Subsequently, the Log10-transformed intensities termed as protein abundance were used for further analysis, resulting in a protein abundance matrix (M) containing 17,951 proteins originating from 7330 different samples (17,951 rows × 7330 columns).

Here, we assumed that the expression of proteins within a protein complex may exhibit concordance, which can be evaluated using the Manhattan distance of their expression abundances. Therefore, we calculated the predefined protein–protein interaction score for a protein pair using the following formula:

(1) $$D_{i,j}=\left|M_{i,.}-M_{j,.}\right|$$

where ($D_{i,j}$) represents the predefined PPI for protein pair *i* and *j*, and $M_{i,.}$ and $M_{j,.}$ are the rows of matrix *M*, which correspond to the abundance of protein *i* and protein *j* across all samples, respectively. The protein abundance feature matrix was calculated for all protein pairs in the training and test sets, generating a 109,988 (protein pairs) (14,186 positive + 95,802 negative) × 7330 protein abundance feature matrix for the training set and a 116,836 (protein pairs) (5781 positive + 111,055 negative) × 7330 protein abundance feature matrix for the test sets.

#### 4.2.2. Protein Interaction Features

For each protein pair in the training and test sets, the AP–MS/CF–MS features comprised 258 features (Table S1) that were generated by integrating over 9000 mass spectrometry experiments from three published papers (Wan et al. [12], BioPlex [5,46], and Hein et al. [8]) [13], which were downloaded from the hu.MAP database (termed protein interaction data in this study) [47]. More specifically, these features were collected from the following 6 resources: (1) 220 co-fractionation features, i.e., 4 types of co-fractionation measures (Poisson noise Pearson correlation coefficient, a weighted cross-correlation, a co-apex score, and a MS1 ion intensity distance metric) for each of the 55 fractions in Wan et al. [12]; (2) nineteen genomic/proteomic/literature features of worm, fly, human, and yeast from HumanNet [48], such as genetic interactions, results of high-throughput yeast 2-hybrid assays, co-citation of genes, et al.; (3) two features that describe protein interactions obtained from AP–MS experiments in fruit fly (“ext_Dm_guru”, [49]) and human (“ext_Hs_malo” [50]); (4) nine features from the BioPlex database, being the NWD score, Z score, plate Z score, entropy, unique peptide bins, ratio, total PSMs, ratio total PSMs, and unique to total peptide ratio; (5) four features from Hein et al., being the Pearson’s correlation coefficient of the intensity profiles of the prey and bait proteins (“prey.bait.correlation”), the number of available quantitative data of the prey (“valid.values”), the log10-transformed of prey-to-bait protein in the pulldown samples (“log10.prey.bait.ratio”), and the log10-transformed of prey-to-bait protein in the HeLa proteome samples (“log10.prey.bait.expression.ratio”) [8]; and (6) four features (i.e., “neg_ln_pval”, “pair_count”, “hein_neg_ln_pval”, and “hein_pair_count”) generated based on Drew et al.’s weighted matrix model interpretation [13] of the AP–MS datasets in BioPlex [5] and Hein et al. [8]. This resulted in a 109,988 (14,186 positive + 95,802 negative) × 258 protein interaction feature matrix and a 116,836 (5781 positive + 111,055 negative) × 258 protein interaction feature matrix for training and test sets, respectively.

### 4.3. Deep Learning Neural Network Implementation

The neural network model was implemented by using the R (version: 3.5.1) interface to Keras (version number: 2.2.5.0), which is a high-level neural network API [51]. Our model consisted of three densely connected hidden layers with different numbers of neurons and the output layer was aimed at predicting PPIs (Figure 1). The rectified linear unit (ReLU) activation functions were used for all hidden layers. The sigmoid activation function was applied to the output layer. For each of the hidden layers, a dropout layer was appended to avoid overfitting. The training process was performed for 10 epochs using the “RMSProp” [52] optimizer with binary cross-entropy as the loss function. The optimal combination of 6 hyper-parameters (the number of neurons in each hidden layer and dropout rate in each dropout layer) were tuned by random searching. Briefly, the number of neurons for each hidden layer was generated randomly, ranging from 10 to 750, and the probabilities for the dropout layers followed a uniform distribution over the interval of 0 to 0.5 (Table S2). We applied this training process on three different feature matrices: (i) protein pairs containing only the protein abundance features, (ii) protein pairs with only the protein interaction features, and (iii) protein pairs that integrated both abundance and interaction features. Subsequently, the F1-measure ($F1\;\mathrm{measure}=2\cdot \frac{precision\;\cdot \;recall}{precision\;+\;recall}$) that represents the harmonic means of precision and recall of the prediction by these models was used to compare the model performance and select the best model. Finally, the best model was applied to all protein pairs with protein abundance and interaction features to generate the weighted PPI network, in which nodes were proteins, and the weight of the edge was the protein–protein interaction probability predicted by the best model.

To evaluate the performance of our model further, we used the interaction feature matrix as input to train the SVM classifiers. The SVM implementation of the R package “e1071” (version 1.7.2) [53], which is based on the LIBSVM library [54], was applied with the function *tune.svm*. To seek an optimal model, we performed a parameter tuning of the hyperparameters (C and gamma) for the SVM model training using 10-fold cross-validation by the *tune.control* function (parameters are detailed in Table S2). The performance of the SVM models was subsequently evaluated by comparing the F1-measures.

### 4.4. Evaluation of Feature Importance

The contribution of features is an important aspect of deep learning. In this study, we utilized Breiman’s feature permutation method to assess feature importance [55]. The original concept involves measuring a feature by calculating the increase in the model’s prediction error after shuffling the feature, which disrupts its relationship with the true outcome. In our study, we evaluated feature importance by observing the decrease in model performance when we randomly shuffled the values within the features. Firstly, the best deep learning model was applied to the test dataset ($T_{0}$) to make the prediction and to calculate the F1-measure ($F1_{0}$). Secondly, only the values of the *i*th feature in the test dataset were randomly shuffled, generating a sudo-test dataset ($T_{i}$), which was fed into the model to make predictions and to calculate the F1-measure ($F1_{i}$). Thirdly, the second step was repeated *N* (*N* = 50) times, and the mean value of the F1-measure for the i_th_ feature ($\frac{1}{N}\sum_{j}^{N}F1_{i,j}$) was calculated. Lastly, the importance of the i_th_ feature ($I_{i}$) was evaluated by

(2) $$I_{i}=F1_{0}-\frac{1}{N}\sum_{j}^{N}F1_{i,j}$$

### 4.5. Two-Stage Clustering to Predict Protein Complexes

The weighted protein interaction network as generated by the final deep learning model using both protein abundance and interaction features was used to derive protein complexes through a two-stage clustering approach. The first clustering method, ClusterOne (clustering with overlapping neighborhood expansion), is a graph clustering algorithm [30], which starts from a single seed vertex and exploits a greedy procedure that adds or removes vertices to find clusters with high cohesiveness. The parameter “density” was set to determine the complex density. The “overlap” specifies the maximum allowed overlap between two clusters, which determines whether to merge or not merge highly overlapping complexes. The second clustering method is MCL (Markov cluster) algorithm) [31]. This unsupervised cluster algorithm is based on stochastic simulation of flow in networks/graphs and is controlled by the inflation (-I) parameter. Inflation affects the granularity or resolution of the clustering outcome, where low values lead to fewer and larger clusters, and high values lead to more and smaller clusters.

We first sorted the edges in descending order by their weights, which were predicted by the deep learning model, resulting in a subnetwork with the top r percent of edges. Here, r (ranging from 1 to 20) is a tuning parameter that needed to be optimized to obtain the best set of complexes in the following steps. In the ClusterOne clustering step [30], a seed method of “nodes” and a minimum size of 2 were applied to each subnetwork (r = r_i_) to generate a set of intermediate clusters. Here the parameters for the ClusterOne algorithm “density” were tuned in the range of [0.2, 0.25, 0.3, 0.35, and 0.4], and “overlap” was tuned in the range of [0.6, 0.7, and 0.8]. Since we allowed merging high-overlapping clusters in the ClusterOne process, this could have led to large clusters that were over-merged, i.e., biologically unrelated complexes merged into a single large cluster [12]. Therefore, a second clustering stage, based on the Markov cluster (MCL) algorithm, was performed on each cluster generated by ClusterOne to split the over-merged clusters. Here, the parameter inflation (-I) of the MCL algorithm [31] was tuned in the range of [1.2, 2, 3, 4, 5, 6, 7, 8, 9, 10, 11, 13, and 15]. Proteins that did not share any edge with the remaining proteins in the final clusters were removed. This two-stage clustering process was carried out for each combination of parameters, i.e., r, density, overlap, and inflation, followed by a k-clique evaluation (the parameter combinations are detailed in Table S3).

### 4.6. K-Clique Method-Based Accuracy Evaluation

To measure the accuracy of the reconstructed complexes, we used the k-clique algorithm for each of the two-stage-clustering results. As described above [13], this approach is based on the matching of cliques within the set of all possible cliques between reconstructed or predicted complexes and benchmark (golden dataset) complexes (here the CORUM complexes). Specifically, the predicted complexes and CORUM complexes were first divided into different subsets according to their clique size *k* (e.g., *k* = 2, all pairwise combinations; *k* = 3, all triplet combinations; etc.). Secondly, we removed the predicted complexes in which all protein members were not in the gold-standard set. In other words, we only evaluated the complexes containing proteins that form known complexes to not penalize novel predicted complexes as false positives. Thirdly, for each clique size *k*, the true positive (TP*_k_*) was defined by the number of common complexes between the predicted complex set and gold-standard complex set; the false positive (FP*_k_*) was the number of complexes in the predicted complex set but not in the gold-standard complex set; and the false negative (FN*_k_*) was the number of complexes in the gold-standard complex set but not in the predicted complex set. Subsequently, the precision (P*_k_*), recall (R*_k_*), and F-measure (F*_k_*) were defined as follows:

(3) $$P_{k}=\frac{{\mathrm{TP}}_{k}}{{\mathrm{TP}}_{k}+{\mathrm{FP}}_{k}}$$

(4) $$R_{k}=\frac{{\mathrm{TP}}_{k}}{{\mathrm{TP}}_{k}+{\mathrm{FN}}_{k}}$$

(5) $$F_{k}=2\times \frac{P_{k}\times {\;R}_{k}}{P_{k}+{\;R}_{k}}$$

Finally, a global F-measure (F-grand, Equation (4)) was defined as the mean of F*_k_*, iterating over clique sizes of *k* from 2 to K, where K is the largest cluster size of the predicted complexes set.

(6) $$F_{\mathrm{grand}\;=}\frac{\sum_{k=2}^{K}F_{k}}{K-1}$$

### 4.7. Enrichment Analysis and Tissue Specificity

We used the g:Profiler web tool [56] to perform protein and pathway enrichment analysis for each predicted complex, with significantly enriched terms (Benjamini-Hochberg FDR < 0.05). For comparing tissue specificity, we mapped our predicted complexes to the tissue-based map of the human proteome from the Human Protein Atlas [32,57].

## Data Availability

The source code and datasets are available at https://github.com/Bohui2447/ProteinComplex1.

## Supplementary Materials

The following supporting information can be downloaded at: https://www.mdpi.com/article/10.3390/ijms24097884/s1. References [58,59,60,61,62,63,64,65] are cited in the supplementary materials.
