# Supplementary material for: Identification of Protein Complexes by Integrating Protein Abundance and Interaction Features Using a Deep Learning Strategy
Source: Int J Mol Sci. 2023 Apr 26;24(9):7884. doi: 10.3390/ijms24097884 (PMC10178578; doi:10.3390/ijms24097884)
Supplement: Supplementary file 1 [file ijms-24-07884-s001.zip › Supplementary.pdf]

Supplementary to "Identification of protein complexes by integrating protein abundance and interaction features using a deep learning strategy"

Protein abundance feature contributes to capturing novel subunits: We predicted that MTF2 (also known as PCL2) is a subunit of the polycomb repressive complex 2 (PRC2) (Fig. S4A, B). The PRC2 core complex, consisting of SUZ12, EED, and EZH2 [1, 2], is important in chromatin compaction and catalyzes the methylation of histone H3 at lysine 27 [3, 4]. We found that the novel member MTF2 showed a high concordance of abundance with the PRC2 core in a comparable set of samples (Fig. S4B). It is reported that PCL proteins, including PCL1, PCL2 (MTF2) and PCL3, interact with PRC2 through EZH2, and to some extent through SUZ12 [5]. Interestingly, we captured this novel interaction, i.e., MTF2-EED, through the interaction of MTF2 with the PRC2 complex, which is also supported by the interaction features, albeit relatively weak, in the study by Hein et al. (Fig. S4A right).

Another example is the complex centralspindlin, reported as a heterotetramer consisting of a dimer of the kinesin KIF23 (also known as MKLP1) and a dimer of the accessory protein RACGAP1 (also known as Cyk4 or MgcRacGAP) [6]. The SHC SH2-domain binding protein 1 (SHCBP1) was predicted by our method as a novel subunit that interacts with the centralspindlin complex through RACGAP1 (Fig.S4C, D). These proteins were detected in 30% (~ 2200) of total abundance samples (Fig. S4D), indicating their co-expression and co-occurring characteristics. Interestingly, interactions between SHCBP1 and RACGAP1 were detected multiple times in the AP-MS interaction map by Hein et al. (Fig. S4C right). These results suggest that incorporating protein abundance features from diverse datasets could improve the prediction of protein complexes and enable the identification of novel interactions with high confidence.

The co-expression feature assists the identification of protein complexes: In addition to the IPO7- KPNB1 complex, we observed that two chaperone proteins VCP (also known as p97) and HSP90B1 (also known as gp96 or GRP94) displayed a high concordance in expression across a great number of the protein abundance samples (Fig. S4E). It has been reported that VCP cooperates with diverse partner proteins to help process ubiquitin-labelled proteins for recycling or degradation by the proteasome in many cellular contexts [7]. Interestingly, the gp96 was also demonstrated governing protein ubiquitination and degradation [8]. Thus, we deduce that p97 and gp96 may interact in their regulation of protein ubiquitination and degradation in the endoplasmic reticulum.

1. Cao, R., et al., *Role of histone H3 lysine 27 methylation in polycomb-group silencing.* Science, 2002. **298**(5595): p. 1039-1043.
2. Czermin, B., et al., *Drosophila enhancer of Zeste/ESC complexes have a histone H3 methyltransferase activity that marks chromosomal polycomb sites.* Cell, 2002. **111**(2): p. 185-196.
3. Margueron, R. and D. Reinberg, *The Polycomb complex PRC2 and its mark in life.* Nature, 2011. **469**(7330): p. 343-349.
4. Laugesen, A., J.W. Hojfeldt, and K. Helin, *Molecular Mechanisms Directing PRC2 Recruitment and H3K27 Methylation.* Molecular Cell, 2019. **74**(1): p. 8-18.
5. Nekrasov, M., et al., *Pcl-PRC2 is needed to generate high levels of H3-K27 trimethylation at Polycomb target genes.* Embo Journal, 2007. **26**(18): p. 4078-4088.
6. Mishima, M., S. Kaitna, and M. Glotzer, *Central spindle assembly and cytokinesis require a kinesin-like protein/RhoGAP complex with microtubule bundling activity.* Developmental Cell, 2002. **2**(1): p. 41-54.
7. Meyer, H., M. Bug, and S. Bremer, *Emerging functions of the VCP/p97 AAA-ATPase in the ubiquitin system.* Nature Cell Biology, 2012. **14**(2): p. 117-123.
8. Wu, B., et al., *Heat shock protein gp96 decreases p53 stability by regulating Mdm2 E3 ligase activity in liver cancer.* Cancer Letters, 2015. **359**(2): p. 325-334.

Supplementary figure captions:

Figure S1. ROC plot

A comparison of ROC for the deep learning and SVM models based on different data sources. The blue line represents the best DL model using integrated protein abundance and protein interaction features, the red dashed line represents the best DL model using protein interaction features, the green dashed line is the best DL model using protein abundance features, and the purple dashed line represents the best SVM model using the protein interaction features. The area under the curve (AUC) of these models are 0.9, 0.86, 0.86, and 0.88, respectively.

Figure S2. Comparison of model performance

The precision is calculated by true positive / (true positive + false positive), and the recall is calculated by true positive / (true positive + false negative). A harmonic mean of precision and recall, namely F1-measure or F1-score was furtherly used to decide the model performance.

A Precision-recall curve of the 63 protein interaction feature matrix based deep learning models, the curve highlighted in red is the best model with F1-measure at 0.61.

B The training of 87 deep learning models with protein abundance features and an F1-measure > 0.49 were used to represent in the precision-recall plot, the curve highlighted in red is the best model with F1-measure at 0.51.

C Precision-recall curve indicating 109 integrated deep learning models with an F1-measure > 0.66, the outperforming model is shown in red with F1-score at 0.68.

D A total of 28 SVM models were trained with the protein interaction features, the red line shows the best SVM classifier with F1-measure at 0.64.

Figure S3. Evaluation of predicted protein-protein interactions

A-C, Scatterplot showing the abundance and interaction as obtained from Hein et al. experiment with different percentage of interaction from our predicted PPI network. A weaker interaction was observed when decreasing the protein interaction confidence, suggesting the importance of this filtering step in obtaining an optimal PPI network.

Figure S4. Protein Complexes with novel subunits as well as newly predicted highly co-expressed protein complexes that are predicted by our model

A left panel; Interaction network of the core Polycomb repressive complex 2 (PRC2, highlighted in blue) with a potential novel subunit MTF2 (purple) as predicted by our model, **A right panel**; Scatter plot with interaction and abundance for the PRC2 complex from Hein et al' AP-MS experiments. Blue dots are known interactions, the purple dots are novel interactions. Labels for the dots are represented by Bait Prey proteins. The predicted EED-MTF2 interaction shows a correlation with the other proteins from the PRC2 complex albeit with lower protein abundance and interaction. Suggesting an interaction between EED-MTF2 albeit a somewhat weaker.

B The expression pattern of each subunit within the PRC2 protein complex. On each row, the X-axis indicates 7,330 samples collected from PRIDE repository and the Y-axis indicates the log10 transformed intensity of corresponding protein, where missing values are in blanks. This plot shows that the expression pattern of MTF2 shares a high concordance with the subunits of the PRC2 complex, suggesting the abundance of proteins could improve the model sensitivity.

C left panel; Interaction network of the Centralspindlin complex (core members are in blue and new predicted protein is in purple). **C right panel**; Interaction-abundance plot for Centralspindlin

complex from Hein et al' AP-MS experiments. Blue dots are known interactions, purple dots are novel interactions. Labels for the dots are represented by Bait_Prey proteins.

D The expression pattern of each subunit within the Centralspindlin protein complex. On each row, the X-axis indicates 7330 samples collected from the PRIDE repository and the Y-axis indicates the log10-transformed intensity, and missing values are in blanks. It can be seen that the novel subunit SHCBP1 shows a high similarity of expression pattern with the subunits of the Centralspindlin complex, suggesting a high possibility of co-occurrance for these proteins.

E Interaction network and protein expression pattern plot for the predicted complex VCP-HSP90B1. These two proteins displayed a high concordance in expression across around 5700 of the protein abundance samples, however, it has not been detected in Hein et al' AP-MS experiment also showing the sensitivity of our deep learning model and the importance of integrating the protein abundance features in protein complex predictions.

Figure S5. Protein complex members show a significant co-expression.

A Our model predicts the interaction network of the 11 subunits multi-synthetase protein complex, which is also a well-defined protein complex found in the CORUM database. This indicates our deep learning model possesses a high accuracy and robustness.

B The expression pattern of the multi-synthetase protein complex. The X-axis indicates 7330 samples collected from PRIDE repository. The Y-axis indicates the log10 transformed intensity of protein, missing values are in blanks. This multi-protein complex contains 11 subunits, which are all co-expressed in most of the samples.

C Interaction network of eukaryotic initiation factor complex with 3 protein subunits.

D The expression pattern of the eukaryotic initiation factor complex. This protein complex contains 3 subunits that are co-expressed in across around 2800 samples, indicating the importance of the co-expression property in predicting protein complexes.

Figure S6. Evaluation of feature importance

A Bar plot shows the importance of features. The importance is indicated by the decrease of F1 measure using the randomly shuffling the values of each feature (see Methods). Red bars represent the protein interaction features and blue the protein abundance features.

B Bar plot shows the average number of proteins in the protein abundance samples with different intervals of importance ranking.

List of supplementary tables

Table S1, The feature information that is used for the training.

Table S1A, This sheet contains all projects that are obtained from PRIDE, with information of project accession, title, project description, publication date, et.al..

Table S1B, This sheet provides the features of the interaction features and corresponding literature.

Table S2, The parameters that are used for the deep learning and SVM models in the training process.

Table S2A, Listed are neuron accounts and dropout parameters for deep learning models with protein interaction features.

Table S2B, This table contains information about neuron accounts and dropout parameters for deep learning models with protein abundance features.

Table S2C, Neuron accounts and dropout parameters for deep learning models using integrated (protein abundance and interaction) features.

Table S2D, Listed are parameters for SVM models with protein abundance features.

Table S3, Parameters used for the ClusterOne (density and overlap) and MCL (Inflation (-I)) clustering and corresponding results of k-clique evaluation.

Table S4, The final set of the predicted complexes, each line indicates one protein complex.
