# Supplementary figures and images for: Identification of Protein Complexes by Integrating Protein Abundance and Interaction Features Using a Deep Learning Strategy
Source: Int J Mol Sci. 2023 Apr 26;24(9):7884. doi: 10.3390/ijms24097884 (PMC10178578; doi:10.3390/ijms24097884)

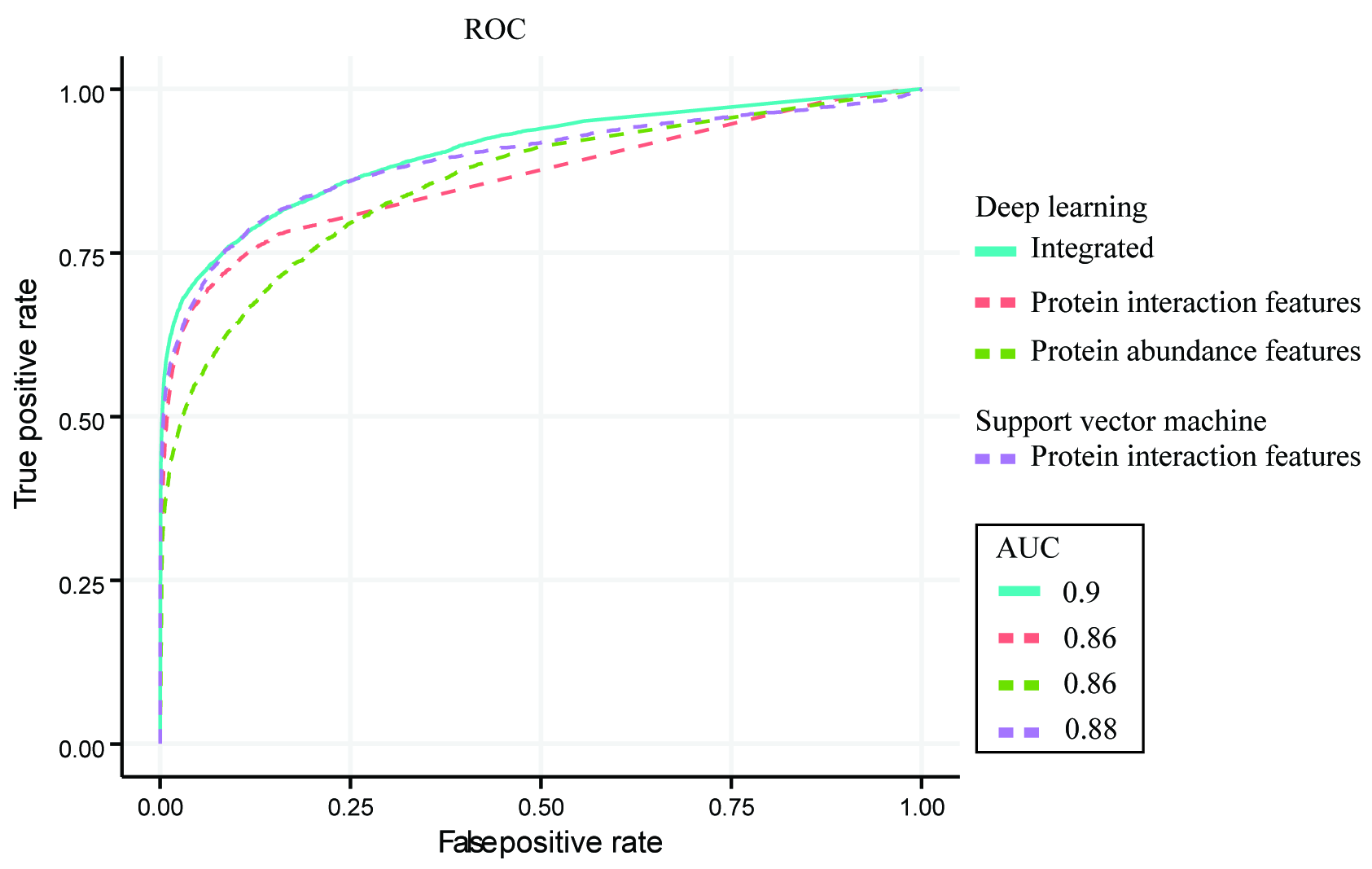

Supplement: Supplementary file 1 [file ijms-24-07884-s001.zip › Figure_S1.tif]

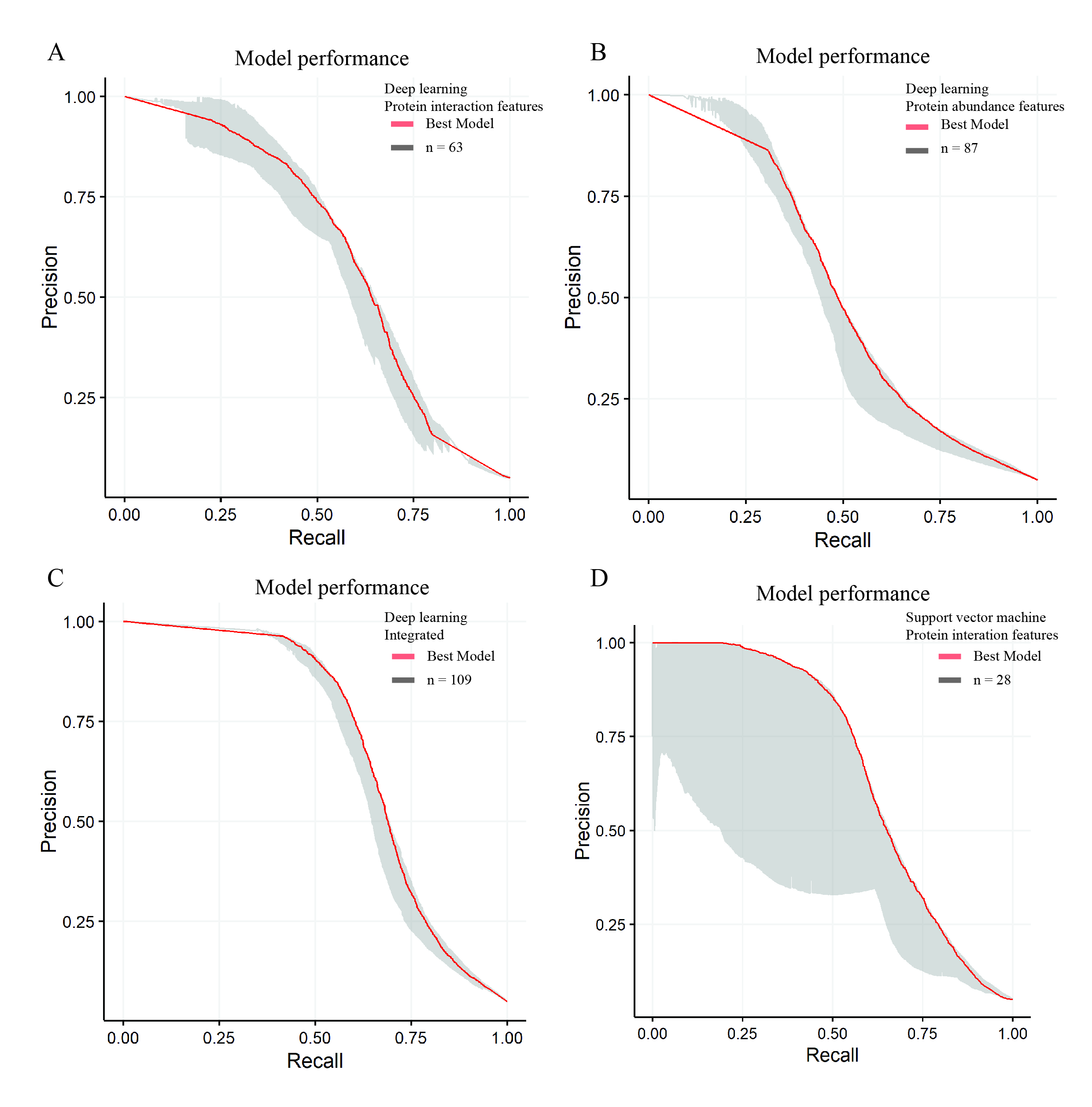

Supplement: Supplementary file 1 [file ijms-24-07884-s001.zip › Figure_S2.jpg]

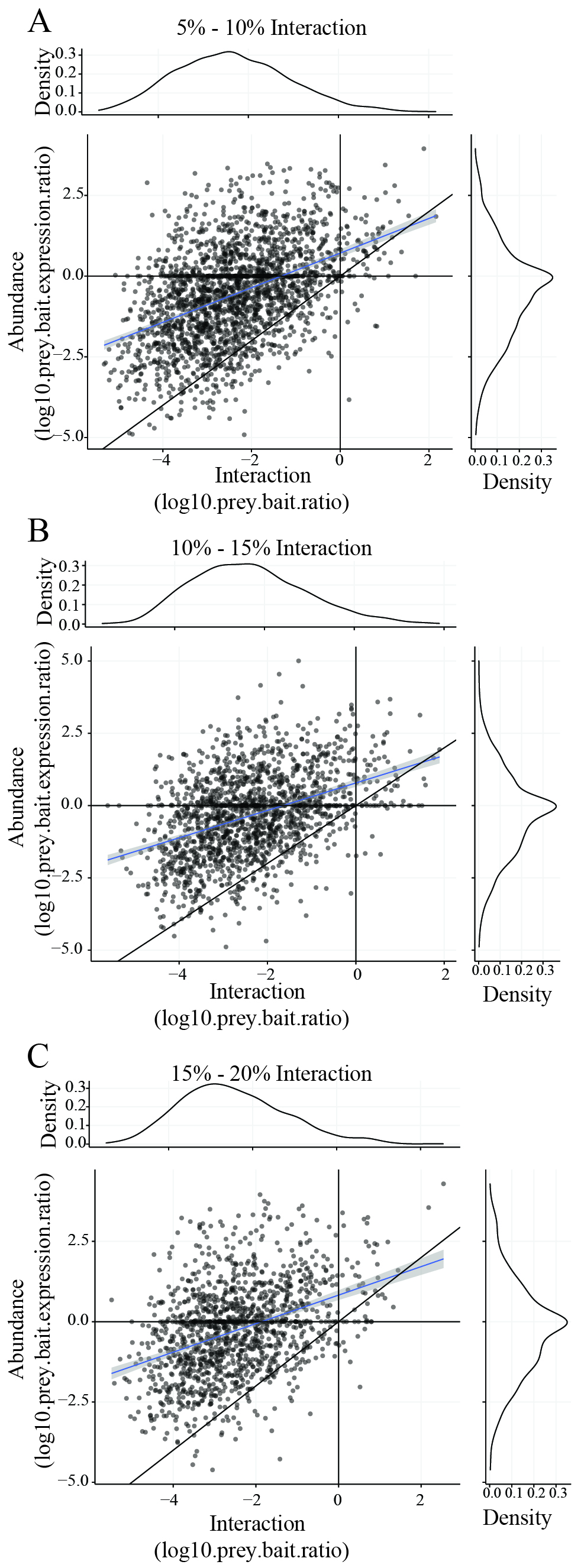

Supplement: Supplementary file 1 [file ijms-24-07884-s001.zip › Figure_S3.jpg]

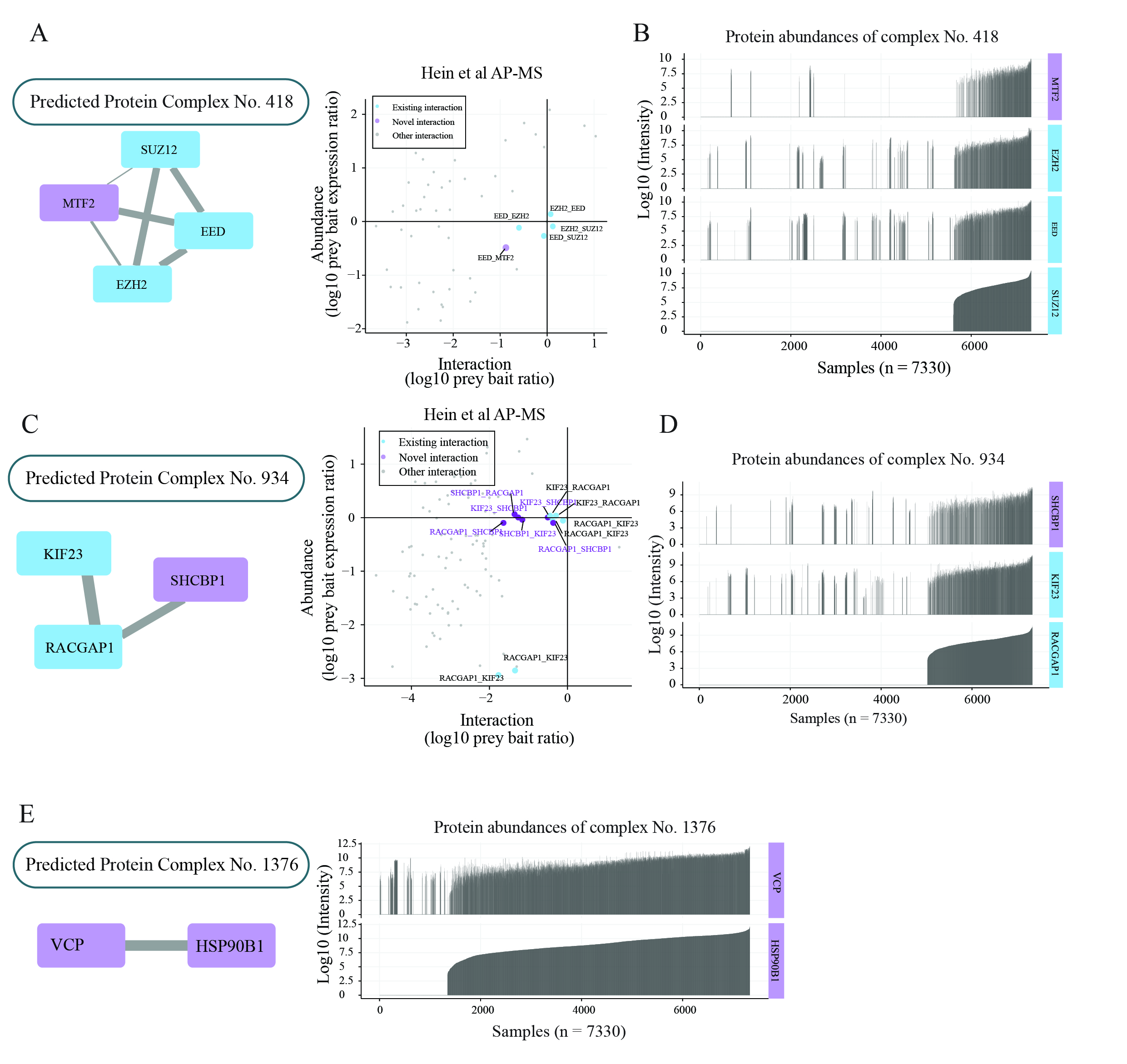

Supplement: Supplementary file 1 [file ijms-24-07884-s001.zip › Figure_S4.jpg]

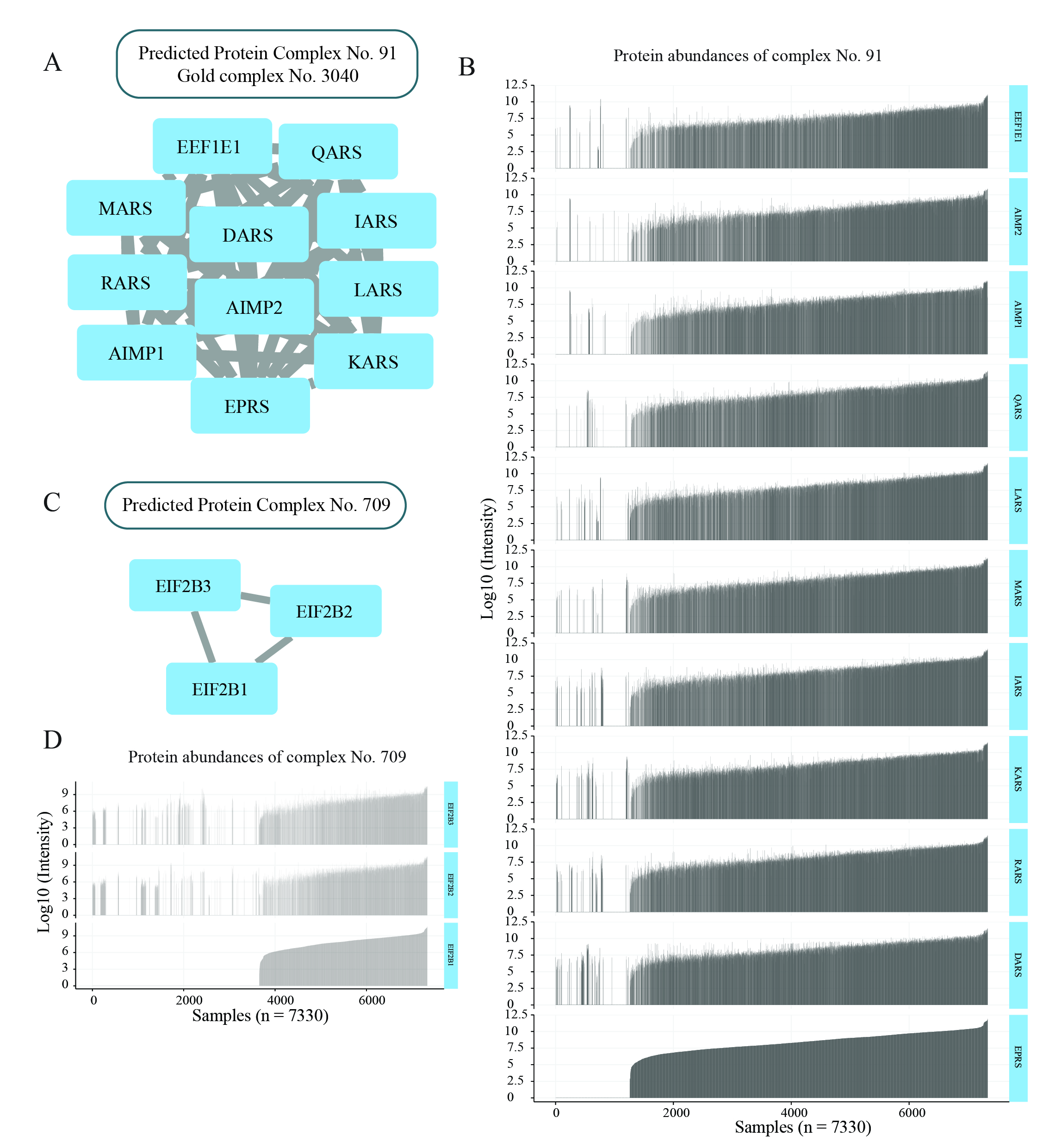

Supplement: Supplementary file 1 [file ijms-24-07884-s001.zip › Figure_S5.jpg]

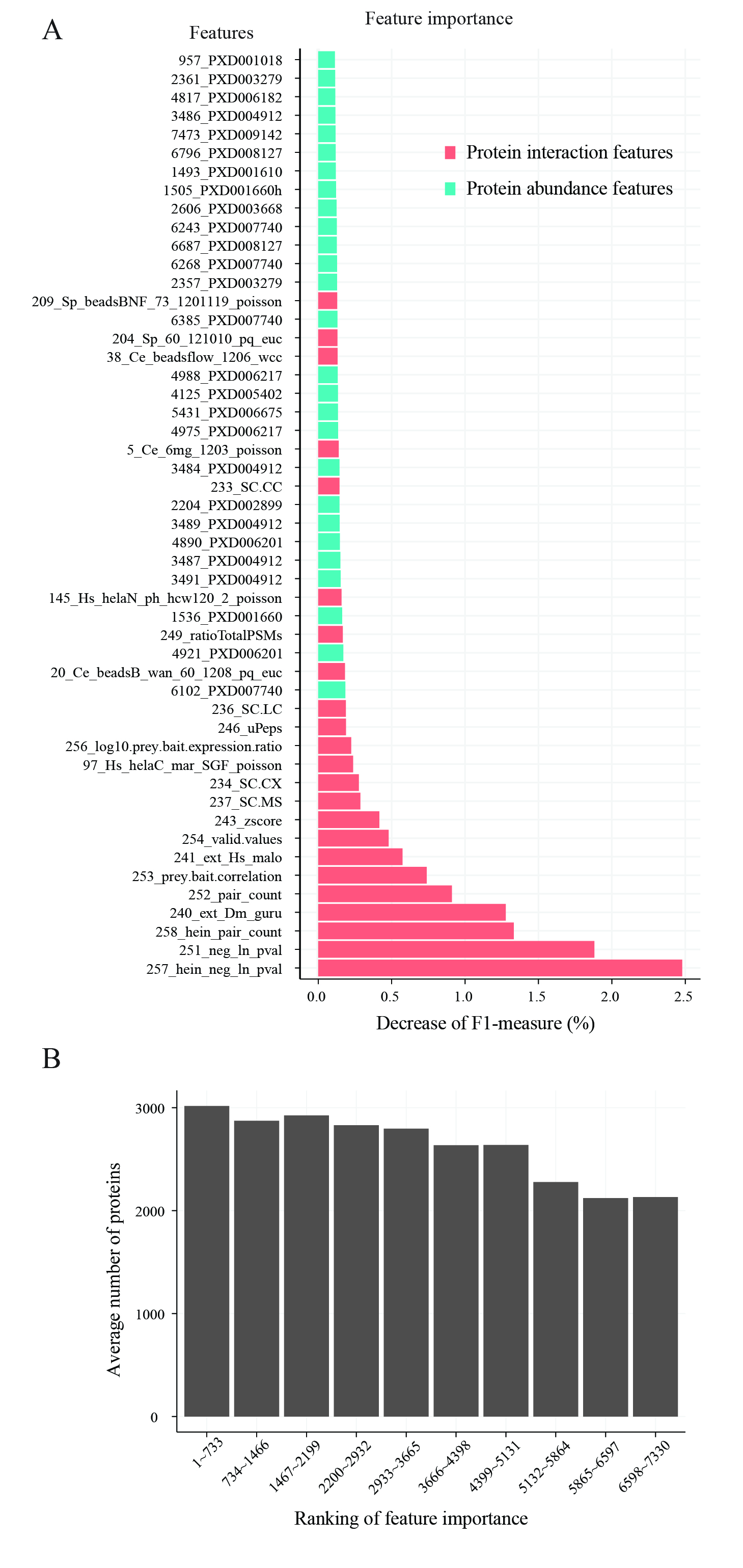

Supplement: Supplementary file 1 [file ijms-24-07884-s001.zip › Figure_S6.jpg]
